# Retrospective case study: ketogenic metabolic therapy in the effective management of treatment-resistant depressive symptoms in bipolar disorder

**DOI:** 10.3389/fnut.2024.1394679

**Published:** 2024-08-12

**Authors:** Nicole Laurent

**Affiliations:** Family Renewal, Inc. DBA Mental Health Keto, Vancouver, WA, United States

**Keywords:** ketogenic diet, bipolar disorder, KMT, ketogenic metabolic therapy, metabolic psychiatry, mood disorders, treatment-refractory depression, clinical psychology

## Abstract

This retrospective case study assessed Ketogenic Metabolic Therapy’s (KMT) efficacy in a bipolar disorder patient with treatment-resistant depressive symptoms insufficiently controlled by weekly ketamine treatments. Monitoring included relevant biomarkers of ketone production and macronutrient levels, alongside mood evaluations through the Generalized Anxiety Disorder-7 (GAD-7), Depression Anxiety Stress Scales (DASS), and PTSD Checklist for DSM-5 (PCL-5), showing mood stabilization and improved functionality. Qualitative analysis revealed sub-stantial enhancements in functioning, life quality, and mental well-being. This study enriches the metabolic psychiatry literature, emphasizing KMT’s potential benefits by integrating quantitative data from recognized psychiatric assessment tools and qualitative insights.

## 1 Introduction

Bipolar II disorder is marked by significant emotional and psychological distress, characterized by periods of depressive episodes and hypomania (1). This condition not only affects an individual’s psychological well-being but also has profound implications on their social and occupational functioning (2). The complexity of Bipolar II disorder, especially with treatment-resistant depressive symptoms, presents a substantial challenge in psychiatric care (3). Current treatments for Bipolar II disorder often include a combination of mood stabilizers, antidepressants, and psychotherapy. However, a notable subset of patients remains resistant to these interventions, experiencing persistent symptoms and a diminished quality of life. Even individuals with bipolar disorder undergoing treatment still spend about 19% of their time in depressive states and an additional 18% in sub-syndromal depressive states (4). This resistance underscores the urgent need for alternative strategies that can offer relief and improve patient outcomes (5).

Emerging evidence suggests that metabolic interventions, such as Ketogenic Metabolic Therapy (KMT), also known as the ketogenic diet, may offer favorable treatment outcomes for individuals with psychiatric disorders. Well established in the management of epilepsy (6), recent studies indicate that the ketogenic diet may have beneficial outcomes for individuals with bipolar disorder, with observations from case studies (7–9) and pilot studies (10–12) reporting notable improvements in symptoms. The diet’s mechanism is believed to involve the modulation of brain energy metabolism and neurotransmitter levels (13–16), providing a compelling rationale for its application in Bipolar II disorder.

This case focuses on an individual diagnosed with Bipolar II disorder, presenting with persistent depressive episodes marked by significant lethargy, low mood, and difficulty in managing daily activities despite standard treatment protocols. By employing both quantitative and qualitative methods, this case study seeks to understand better the treatment potential of KMT with patients for whom standard care has not yielded satisfactory outcomes.

## 2 Case presentation

### 2.1 Clinical background

In this case, a 53-year-old female with Bipolar II reported persistent mood instability and depressive episodes resistant to past and current conventional treatments. Psychiatric intervention at time of diet implementation consisted of weekly ketamine treatments for temporary symptom relief. Despite this intervention, the relief from depressive symptoms was short-lived, lasting only 1 to 3 days before the symptoms returned. The patient also experienced migraine headaches. Prior attempts at management included medication, psychotherapy, a Mediterranean diet, physical exercise, and consistent sleep schedules, which yielded limited improvement. Given the limited efficacy of standard treatments and the transient benefits achieved with ketamine therapy, she was open to exploring KMT as a novel intervention. Her history of psychiatric conditions began in childhood and adolescence, leading to subsequent diagnoses of Generalized Anxiety Disorder and Major Depressive Disorder before the eventual identification of Bipolar II as the most recent diagnosis. At the initiation of treatment, the participant was receiving medical care for additional chronic conditions, which included Immune Thrombocytopenia, Migraines, Hypothyroidism, and recurrent shingles (Herpes Zoster).

### 2.2 Ketogenic metabolic therapy intervention strategy

Macronutrient tracking was initiated using Cronometer, which identified an average baseline carbohydrate consumption of between 200 and 300 g per day. BMI was in a healthy range at diet commencement and remained so throughout treatment. Virtual meetings for KMT support were scheduled twice weekly for 30-min intervals over 3 months and then moved to weekly. Carbohydrate consumption was systematically reduced over 2 weeks to achieve a 30 g total intake per day. Macronutrient ratios were initially set at a 1:1 ratio and later adjusted to a 1.5:1 ratio (154 g Fat, 72 g Protein, 30 g Total Carbohydrates) to increase ketone production. Total carbohydrate measurement was chosen over net to initiate and maintain ketosis at consistent levels. Both ratios used are generally considered Modified-Atkins (MAD). The diet consisted primarily of beef, pork, chicken, eggs, dairy, and salmon, with primary fat sources being MCT oil, avocado oil, and butter. Low-carbohydrate vegetables and minimal amounts of low-carb berries complemented this.

**Figure 1:**
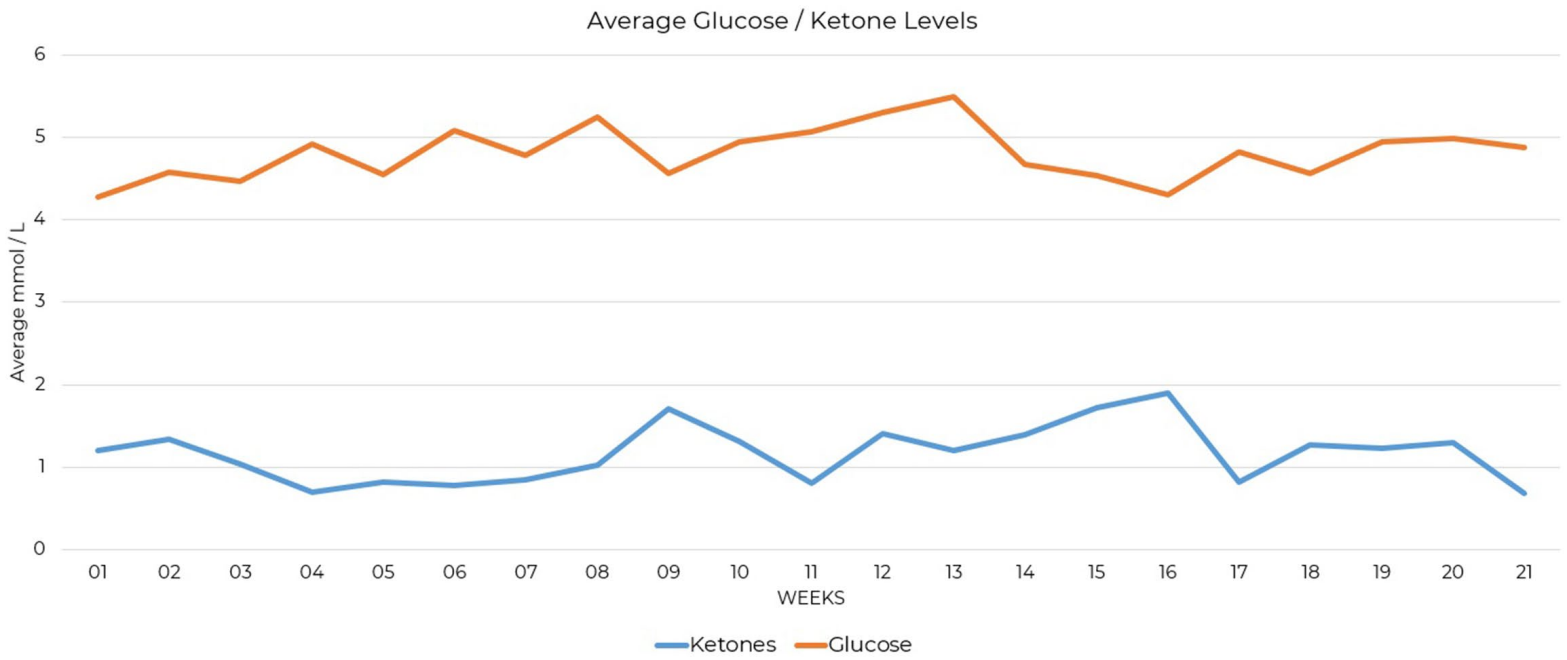
Line graph showing average glucose and ketone levels in mmol/L over 21 weeks.

Supplementation provided included a non-methylated B-complex, trace minerals (providing zinc, copper, manganese, chromium, molybdenum, boron, and vandyl sulfate), vitamin D, and electrolytes in the form of sodium, magnesium, and potassium. Testing compliance was 89% complete for daily ketone measures and 91% complete for daily glucose measures over the 21-week period. Blood glucose and BHB level tracking was initiated and showed nutritional ketosis was achieved at 1.0 mmol/L (Figure 1). Approximately 3.5 weeks into the process of carbohydrate restriction, lab work was received showing free carnitine at 16 μmol/L that identified hypocarnitinemia (17), prompting ongoing L-carnitine supplementation of 3,000 mg in divided doses daily.

## 3 Evaluation of intervention outcomes

### 3.1 Quantitative analysis

Mood assessments were collected at baseline, one-month, four-month, and five-month intervals. They were selected for their validity in assessing self-reported markers of mood, anxiety, stress, and PTSD symptoms. The Generalized Anxiety Disorder-7 (GAD-7), Depression Anxiety Stress Scales (DASS), and PTSD Checklist for DSM-5 (PCL-5) were used. Although no prior diagnosis of PTSD was given, the PCL-5 includes items that assess symptoms such as trouble sleeping, feeling easily startled, difficulty concentrating, and strong negative emotions, which can overlap with symptoms of Generalized Anxiety Disorder, Major Depressive Disorder, and Bipolar Disorder. As the case study participant had received these diagnoses in the past, its inclusion allowed for the detection of nuanced symptom changes potentially relevant in measuring changes in mental health status.

**Figure 2:**
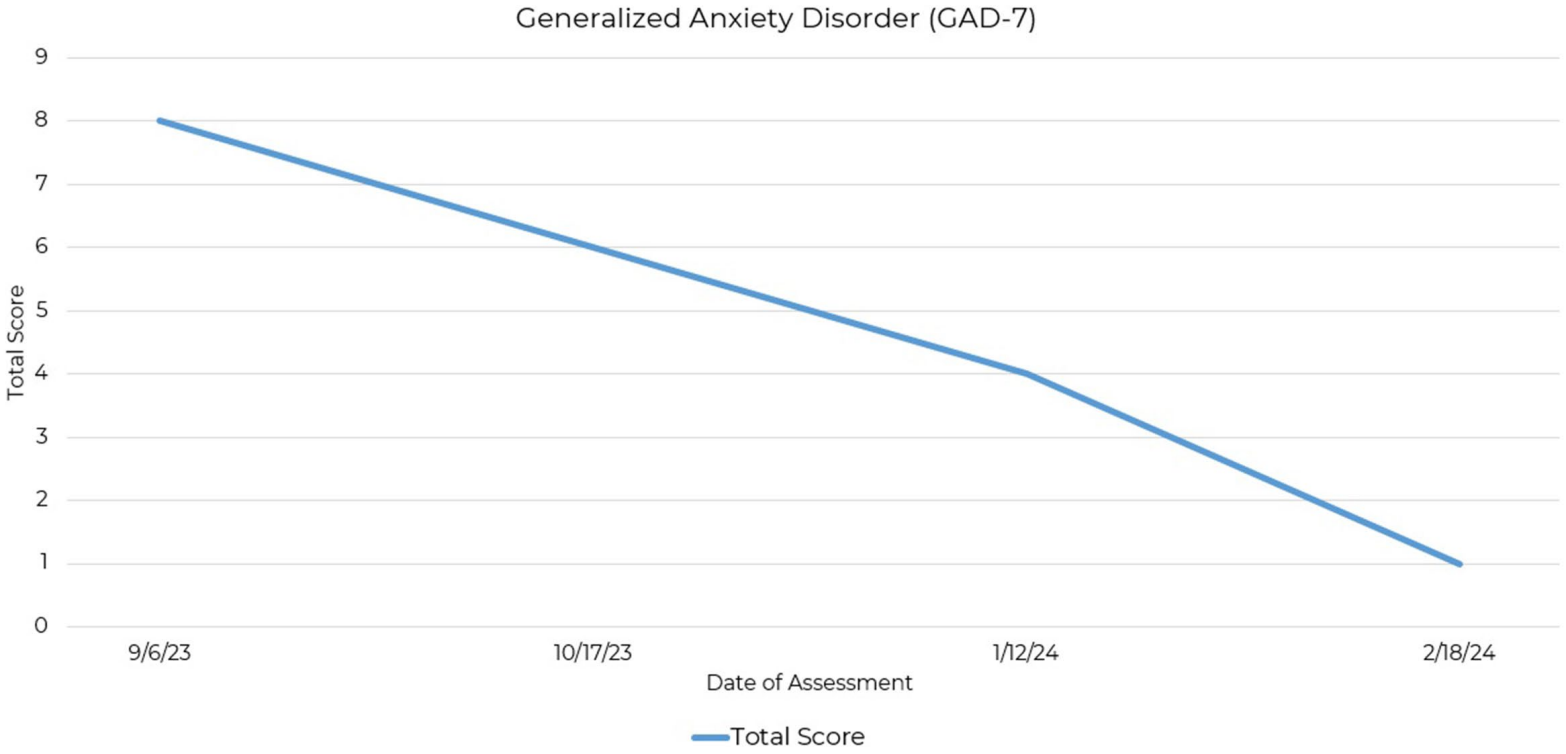
Line graph depicting the reduction in GAD-7 total scores across four assessment points over a 21-week period.

The Generalized Anxiety Disorder-7 (GAD-7) is a self-reported assessment measuring the severity of anxiety symptoms and is considered a dimensional indicator of Generalized Anxiety Disorder severity (18). Scores at the onset indicated mild symptoms, which decreased over the course of the intervention, ending in a normal range (Figure 2). A breakdown of these changes is presented (Supplementary Table S1), quantifying the initial severity and subsequent reductions in GAD-7 scores over the 21-week period.

**Figure 3:**
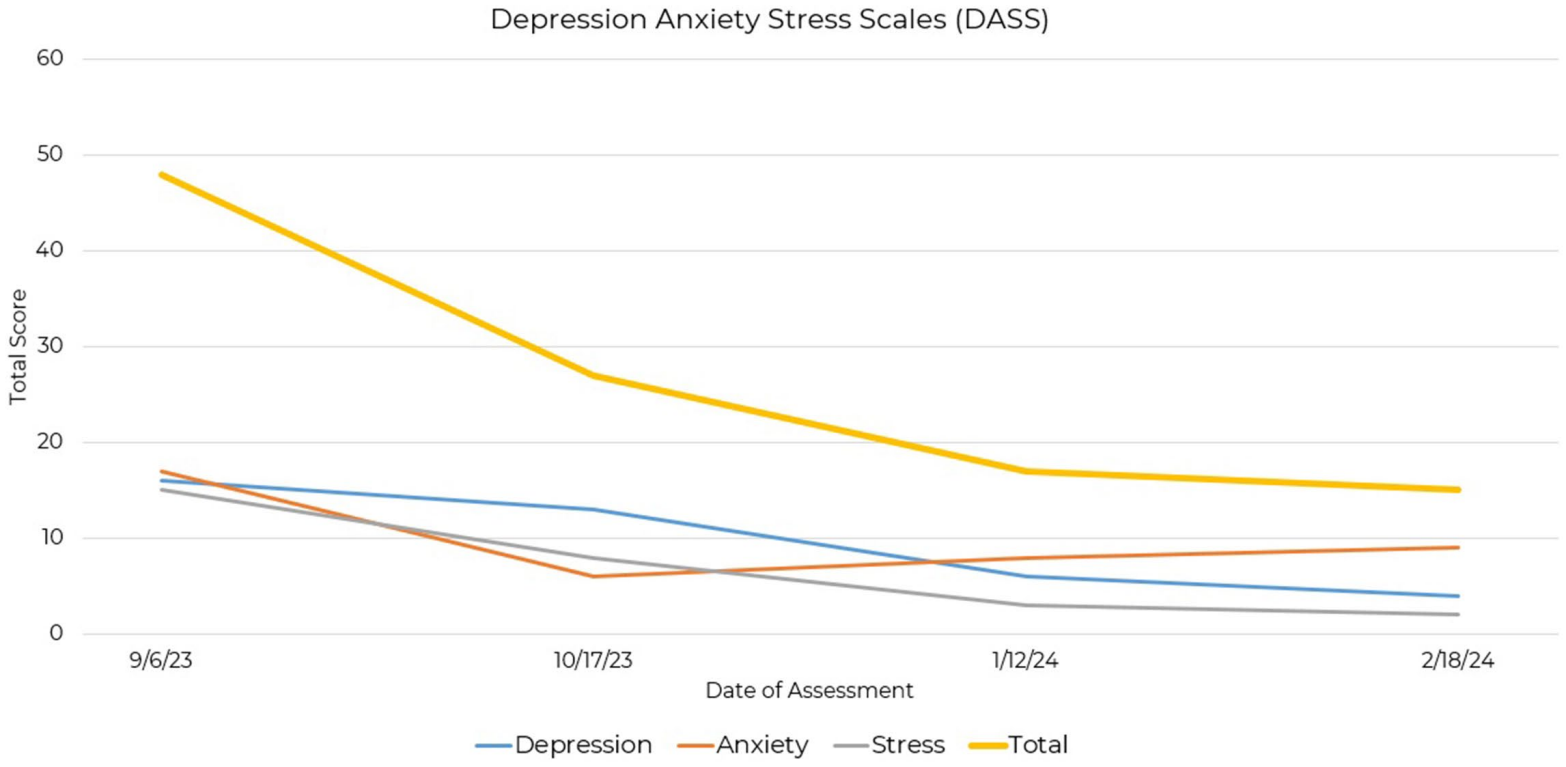
Line graph depicting the reduction in DASS total and subscale scores across four assessment points over a 21-week period.

The Depression Anxiety Stress Scales (DASS) is based on a dimensional rather than a categorical conception of psychological disorders and differentially assesses three negative emotional states: depression, anxiety, and stress (19, 20). Initial evaluations showed high levels of these symptoms, especially depression, indicating substantial emotional distress. The 42-item version of the DASS was administered with scores indicating a reduction in symptoms (Figure 3).

Baseline scores indicated moderate to severe levels of depression, anxiety, and stress, with reductions across all three subscales as treatment progressed. Particularly notable was the decrease in depression scores from a moderate level to a normal range. Additionally, anxiety and stress scores showed decreases, indicating a shift towards milder symptomatology (Supplementary Table S2). Differences in initial severity scores between the GAD-7 and DASS anxiety scale could be attributed to the broader assessment coverage provided by the DASS.

**Figure 4:**
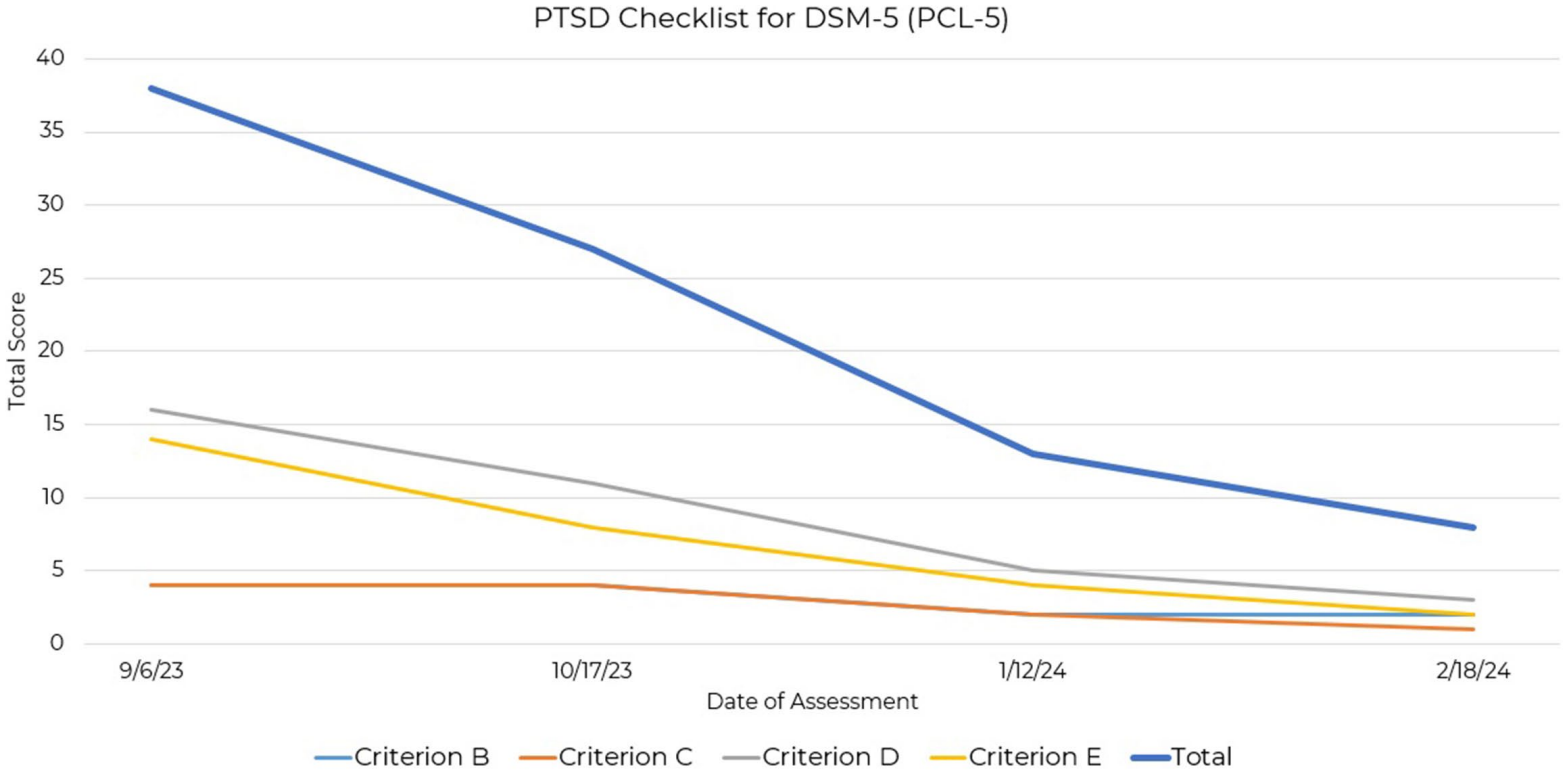
Line graph depicting the reduction in PCL-5 total and criterio subscales across four assessment points over a 21-week period.

The PTSD Checklist for DSM-5 (PCL-5) is a self-report rating scale for assessing the 20 DSM-5 symptoms of post-traumatic stress disorder (21). Initial assessment revealed endorsement of Criterion D (negative alterations in cognitions and mood), initially exhibiting the highest severity, and Criterion E (alterations in arousal and activity). Subsequent assessments showed a consistent decrease in these scores, with marked improvements observed in both Criterion D and Criterion E (Figure 4).

Although there are currently no empirically derived severity ranges for the PCL-5 (22), reductions in Criteria D and E suggest improvement in mood and arousal symptoms over the assessment period. These criteria, indicative of symptoms seen also in depression and anxiety, may serve as markers of symptom improvement relevant to this case study participant (Supplementary Table S3).

### 3.2 Qualitative analysis

Qualitative analysis, as delineated by Yin and discussed in Baškarada (23), was employed to ensure the systematic collection, analysis, and interpretation of data. The qualitative component of data collection centered on the exploration of participant experience using KMT as a treatment for mental illness, recognizing that quantitative assessments may not fully encapsulate the participant’s experience.

Deductive thematic analysis was applied to the case study’s transcript data, focusing on four predefined themes: the personal and emotional journey with KMT, the adoption decision-making process, enhancements in quality of life, and a comparative analysis of conditions before and after KMT. Open-ended, non-leading questions encouraged unbiased responses, developed in line with the Case Report (CARE) guidelines (24) and as detailed in Supplementary Table S5. Conducted virtually after informed consent, the interview’s structured approach, conducted by the case study author and guided by these themes, facilitated the categorization of the transcript via a systematic coding procedure. Deductive coding in a single case allows focus on specific theoretical constructs that enable a targeted exploration of the participant’s experiences, as detailed in Supplementary Table S4, which links the coding strategy directly to the theoretical constructs addressed. Incorporating peer debriefing and soliciting participant feedback on the interview’s comprehensiveness and preliminary findings helped manage researcher bias, ensuring an objective qualitative examination of KMT’s impact in this single case study analysis.

#### 3.2.1 Personal and emotional journey with KMT

The theme ‘Personal and Emotional Journey with KMT’ was used to identify codes for symptom severity, emotional impact, and personal insights. These codes were utilized to document the participant’s mental and physical health fluctuations, emotional responses, and self-reflections on their experience with KMT, focusing on the direct impact of KMT on the individual’s life. Codes developed within this theme identified the experience of a personal and emotional journey with KMT that communicated the transition from a state of profound mental health struggles to a newfound stability and normalcy. Clinically, this reflected a significant shift in self-perception and emotional regulation, which is foundational in the therapeutic process (25, 26). The narrative revealed how, for this participant, KMT facilitated a re-engagement with life with movement from a position of vulnerability and isolation to one of agency and connectedness. An example of coded data included the patient stating, “I think everyone has to deal with some anxiety and depression. I feel like the amount that I have in my life at this point is like a normal amount.”

#### 3.2.2 Adoption decision-making process

The “Adoption Decision-Making Process” theme and subsequent code development investigated the participant’s route to choosing the intervention. It examined past treatments, differences between expected and actual effects, factors influencing their choice, intervention tolerability, and the potential impact of earlier access. This distillation attempted clarification of the participant’s decision-making framework. Actual codes applied included ‘Previous Treatments,’ ‘Expectations vs. Reality,’ ‘Journey to KMT,’ and ‘KMT Treatment Availability.’

In this single case, the participant’s decision-making process was driven by frustration with standard-of-care treatments towards the adoption of the KMT approach. This identification of a pivotal decision-making phase was suggestive that active patient engagement in treatment choices might be indicative of the broader search for autonomy and efficacy in treatment strategies among individuals with treatment-resistant conditions. The coded narrative identified the psychological impact of finding new hope after numerous failed attempts with traditional therapies and reflected critical moments of self-determination, where the participant took an active role in their KMT treatment plan. The theme adequately captured that the participant viewed the intervention as sustainable with prolonged continuation as needed to control symptoms. The theme was further able to identify an expression of the participant that they would have preferred earlier introduction to the therapy, indicating that the current substantial relief they experienced may not have been achieved had they not discovered this treatment option on their own. This sentiment highlights the importance of early and proactive consideration of KMT by mental health and other professionals with whom they come in contact. An example of coded data included the patient stating, “I do not think if I had not stumbled upon it myself, and had just a very open and caring practitioner to discuss it with for the first time, that I would be experiencing the sense of relief that I’m experiencing today.”

#### 3.2.3 Enhancements in quality of life

Delineating through deductive analysis, the theme of “Enhancements in Quality of Life” focused on capturing the broad improvements in the participant’s life following KMT adoption. This theme encompassed codes for ‘Lifestyle Adjustments,’ detailing changes in habits and routines, and ‘Life Quality Improvement,’ highlighting overall enhancements in life satisfaction across relationships, work, hobbies, and lifestyle. These codes detailed multifaceted benefits beyond clinical symptom alleviation to identify positive impacts on daily living and well-being.

The findings demonstrated improvements in quality of life post-KMT adoption were suggestive of the therapy’s capacity to effect change beyond symptom relief, touching on aspects of daily functioning, social engagement, and overall well-being. Clinically, this theme highlights the impact of KMT, suggesting that its benefits extend into the psychosocial realm, enhancing patients’ ability to engage in meaningful relationships, pursue interests, and maintain a sense of normalcy. The narratives reveal a restoration of hope and vitality, which is paramount in the recovery process. This enhancement in quality of life can possibly be attributed to the stabilizing effects of KMT on mood, which, in turn, facilitates greater emotional resilience and adaptability in facing life’s challenges. An example of coded data included the patient stating, “I actually made the drive with very little fatigue, no anxiety, great energy. All the things that kind of crop up at those kind of appointments happened, but I felt like I dealt with them just so much more easily. Just easily!”

#### 3.2.4 Conditions before and after KMT

The ‘Conditions Before and After KMT’ theme, through deductive analysis, captured the participant’s experiences pre-and post-KMT adoption, employing codes for detailed comparisons and evaluation of efficacy. Codes within this theme included ‘Before After Comparison’ for specific contrasts in conditions and emotional states and ‘Treatment Efficacy’ assessing KMT’s performance against prior treatments. An example of coded data included the patient stating, “I just spent a lot of time very depressed and feeling very withdrawn,” to describe their prior experience. This structured analysis sought to clarify the participant’s experience of the impacts of KMT on their condition and life, offering a more nuanced understanding of KMT’s effectiveness and its role in altering patient outcomes.

The comparative analysis of conditions before and after implementing KMT for this participant provided a clear contrast between the debilitating effects of bipolar disorder and the empowering influence of effective management through this therapy. This theme is clinically significant as it illustrates the potential of KMT to redefine the treatment landscape for individuals with treatment-resistant bipolar disorder. The narrative highlighted a marked improvement in mood stability, cognitive function, and overall well-being, endorsing the effectiveness of KMT in addressing the complex needs of this population. The theme also reflected the broader implications of KMT for clinical practice, framing KMT as a viable approach for the management of bipolar disorder and enhancing patient outcomes.

From a clinical perspective, the analysis of data from this theme underscored the significance of KMT as a possibly viable intervention for individuals with treatment-resistant bipolar disorder. The collected narrative provided a detailed account of KMT’s impact on personal well-being, decision-making processes related to treatment choices, quality of life improvements, and the condition’s comparative state before and after KMT implementation. These findings offer valuable insights into the potential of KMT to augment clinical practice and patient management.

## 4 Discussion

The participant further reported that in response to significant reductions in symptoms and under the guidance of their physician, they were able to discontinue the use of some medications and reduce others previously prescribed for the aforementioned chronic conditions. In regards to mood, initial improvements were verbally reported by the patient 2 weeks after diet initiation. Improvements in mood continued and were generally maintained 5 months following the initiation of KMT, offering data on the timeline of symptom improvement. This data may be helpful for aligning the expectations of both patients and clinicians, as well as for informing the design of future research studies. Studies with extended durations or follow-ups may better capture the potential benefits of KMT as a treatment option for mental illnesses.

This participant’s outcome suggests that metabolic health interventions, like ketogenic diets, could offer new directions for treating psychiatric disorders, especially where standard-of-care treatments fall short. The qualitative analysis suggests the possibility that those suffering from Bipolar II disorder may benefit from early introduction to the treatment as an option. While promising, these findings stem from a single case, urging further research to validate these results in broader clinical settings. This work supports further research on the use of KMT as a potential treatment in psychiatry.

## 5 Conclusion

In this case study, a ketogenic diet significantly improved treatment-resistant depressive symptoms in a patient with bipolar disorder. Both mood assessments and the patient’s experience showed marked improvements. Mood scores moved to normal ranges, indicating stabilized mental health. The patient’s account highlighted improved functioning, better quality of life, and emotional well-being. This case study is of particular interest because it documents the longer-term feasibility of diet implementation, ketone testing compliance, and improvements in relevant symptoms reported by qualitative and quantitative methods. However, any conclusions based on this case study are severely limited by its single-participant sample size and retrospective design, highlighting the need for further research employing randomized controlled trials. Integration of both quantitative and qualitative data may be valuable to adequately represent improvements that researchers are attempting to document as a result of using KMT as a treatment for mental illness.

## Data availability statement

The original contributions presented in the study are included in the article/Supplementary material, further inquiries can be directed to the corresponding author.

## Ethics statement

Ethical approval was not required for the studies involving humans because this was a retrospective case study. After the intervention, the participant decided whether or not they wanted to contribute their experience to the research. Informed consent was obtained to use existing quantitative data and collect case study interview data for analysis. The studies were conducted in accordance with the local legislation and institutional requirements. The participants provided their written informed consent to participate in this study. Written informed consent was obtained from the individual(s) for the publication of any potentially identifiable images or data included in this article.

## Author contributions

NL: Writing – original draft, Writing – review & editing.
