# Supplementary material for: Retrospective case study: ketogenic metabolic therapy in the effective management of treatment-resistant depressive symptoms in bipolar disorder
Source: Front Nutr. 2024 Aug 12;11:1394679. doi: 10.3389/fnut.2024.1394679 (PMC11346312; doi:10.3389/fnut.2024.1394679)
Supplement: Supplementary file 5 [file Table_5.docx]

Supplementary Table 5: Themes, Codes, and Interview Excerpts

This table presents themes, codes, and representative interview excerpts.

| Theme | Codes | Interview Data Excerpt |
| --- | --- | --- |
| Personal and Emotional Journey KMT | Symptom Severity | “I just spent a lot of time very depressed and feeling very withdrawn. I did a lot of isolating from other people, and I spent most of my time almost really, almost all of my time at home. A lot of that time by myself.” |
|  | Emotional Impact | “And then just struggling to think about what I'm gonna do, you know, just really thinking about how bad I feel for a majority of the day. A lot of ruminations. Negative thoughts.” |
|  | Personal Insights | “I think everyone has to deal with some anxiety and depression. I feel like the amount that I have in my life at this point is like a normal amount.” |
| Adoption Decision-Making Process | Previous Treatments | “I had tried several different antidepressants over many years anti anxiety medications like the Benzo class of medications. I tried many alternative healing modalities, such as acupuncture and different type of anti inflammatory nutritional changes, dietary or health changes to try to get a handle on it. Talk therapy. I also did Ketamine.” |
|  | Expectations vs. Reality | “I was, however, extremely surprised to just, to just note how many carbs were in the things that I was used to consuming, and how much I would have to restrict even, you know many vegetables. That was a surprise to me.” |
|  | Journey to KMT | “So I started just learning more about what bipolar 2, what the symptoms of it were, what the different treatments, and she was working with me to explain how the different medications worked, what the side effects, what I could expect. And I just wasn't really sure if I wanted to.” |
|  | KMT Treatment Availability | “I don't think if I hadn't stumbled upon it myself, and had just a very open and caring practitioner to talk, you know, discuss it with for the first time, that I would be experiencing the sense of relief that I'm experiencing today.” |
| Enhancements in Quality of Life | Lifestyle Adjustments | “I'm doing a lot of cooking, that is, that is, taking quite a bit of my time.” |
|  | Life Quality Improvement | "I actually made the drive with very little fatigue, no anxiety, great energy. All the things that kind of crop up at those kind of appointments happened, but I felt like I dealt with them just so much more easily. Just easily!" |
| Conditions Before and After KMT | Before After Comparison | “I just spent a lot of time very depressed and feeling very withdrawn.” |
|  | Treatment Efficacy | “So it's really helped with anxiety, depression, and irritability.” |
